# Genetic engineering of *Arabidopsis* to overproduce disinapoyl esters, potential lignin modification molecules

**DOI:** 10.1186/s13068-017-0725-0

**Published:** 2017-02-17

**Authors:** Shinyoung Lee, Huaping Mo, Jeong Im Kim, Clint Chapple

**Affiliations:** 1 Department of Biochemistry, Purdue University, West Lafayette, IN 47907 USA; 2 Department of Medicinal Chemistry and Molecular Pharmacology, Purdue University, West Lafayette, IN 47907 USA; 3 Center for Plant Aging Research, Institute for Basic Science (IBS), Daegu, 711-873 Republic of Korea

**Keywords:** Phenylpropanoid, Lignin, Sinapoyl, Carboxypeptidase, *Arabidopsis*, Metabolite

## Abstract

**Background:**

Monolignol-like molecules can be integrated into lignin along with conventional monolignol units, and it has been shown that the incorporation of non-canonical subunits can be used to generate hydrolysable lignin by introduction of ester linkages into the polymer and that this type of lignin is more easily removable. Disinapoyl esters (DSEs), which to some degree resemble the monolignol sinapyl alcohol, may be promising lignin modifying units for this purpose. As a first step toward determining whether this goal is achievable, we manipulated metabolic flux in *Arabidopsis* to increase the amounts of DSEs by overexpressing *sinapoylglucose:sinapoylglucose sinapoyltransferase* (*SST*) which produces two main DSEs, 1,2-disinapoylglucose, and another compound we identify in this report as 3,4-disinapoyl-fructopyranose.

**Results:**

We succeeded in overproducing DSEs by introducing an *SST*-overexpression construct into the *sinapoylglucose accumulator1* (*sng1*-*6*) mutant (*SST*-OE *sng1*-*6*) which lacks several of the enzymes that would otherwise compete for the SST substrate, sinapoyglucose. Introduction of *cinnamyl alcohol dehydrogenase*-*c* (*cad*-*c*) and *cad*-*d* mutations into the *SST*-OE *sng1*-*6* line further increased DSEs. Surprisingly, a *reduced epidermal fluorescence* (*ref*) phenotype was observed when *SST*-OE *sng1*-*6* plants were evaluated under UV light, which appears to have been induced by the sequestration of DSEs into subvacuolar compartments. Although we successfully upregulated the accumulation of the target DSEs, we did not find any evidence showing the integration of DSEs into the cell wall.

**Conclusions:**

Our results suggest that although phenylpropanoid metabolic engineering is possible, a deeper understanding of sequestration and transport mechanisms will be necessary for successful lignin engineering through this route.

**Electronic supplementary material:**

The online version of this article (doi:10.1186/s13068-017-0725-0) contains supplementary material, which is available to authorized users.

## Background

The phenylpropanoid pathway generates a variety of metabolites such as flavonoids, salicylic acid, hydroxycinnamate esters, and lignin. Molecular genetic approaches have identified the major genes in this pathway, and the availability of these genes has enabled studies aimed at manipulating the lignin polymer, motivated primarily by its economic importance [27, 48, 52, 53, 56]. Historically lignin engineering has focused on enhancing pulp production and forage digestibility [3, 10, 19, 22, 24, 41]. Recently, this area of research has gained attention in the context of efficient biofuel production from lignocellulosic biomass as a way to enhance energy security and combat global warming [8, 14, 23]. In all cases, the aim of lignin engineering has been to reduce lignin content or change lignin composition/structure in order to minimize the inhibitory effects of lignin on cell wall polysaccharide hydrolysis thereby improving the efficiency of pulp production, animal nutrition, and biofuel synthesis.

The detailed analysis of lignin in a broad range of plants combined with a number of genetic engineering studies suggested a new opportunity to engineer lignin. This strategy is based on the observation that atypical phenylpropanoids can be incorporated as lignin units [24, 30, 37–39, 55]. Normal lignin is polymerized via radical coupling reactions between three kinds of monolignols, *p*-coumaryl alcohol, coniferyl alcohol, and sinapyl alcohol; however, it has become clear that the lignin polymerization machinery can incorporate into the polymer a variety of monolignol-like molecules that are generated in specific genetic backgrounds. For example, monolignol precursors such as coniferaldehyde and sinapaldehyde are accumulated in cinnamyl alcohol dehydrogenase (CAD)-deficient mutants and transgenics and are integrated into the lignin of these plants [4, 20, 40, 47]. The incorporation of 5-hydroxyconiferyl alcohol in caffeic acid *O*-methyltransferase mutants [24, 30, 38, 39], caffeyl alcohol in a caffeoyl CoA 3-*O*-methyltransferase mutant [55], and ferulic acid in a cinnamoyl CoA reductase mutant [37] provides additional examples. Further integration of various phenylpropanoids into lignin and altered lignin characteristics have been clearly demonstrated using in vitro lignification assays or the maize cell wall system [16, 18, 51]. Enhanced cell wall degradability could potentially be achieved by manipulating lignin in several different ways: (1) reducing hydrophobicity by incorporating phenolics with hydrophilic residues such as feruloylquinic acid and guaiacyl glycerol, (2) reducing cross-links between lignin and cell wall polysaccharides by introducing phenolics such as caffeates and catechins with *o*-diphenol functionality, and (3) integrating phenolics such as coniferyl ferulate and rosmarinic acid with easily hydrolysable ester bonds. *In planta,* success has been achieved by overexpressing bacterial hydroxycinnamoyl-CoA hydratase-lyase, which causes the integration of side-chain-truncated lignin monomers into lignin and the reduction in the degree of lignin polymerization [12] and most recently in the case of expression of feruloyl-CoA coniferyl alcohol feruloyltransferase in transgenic poplar [58].

In this study, we focused on enhancing the production of disinapoyl esters (DSEs) for future use as lignin modification subunits. Two DSEs have been identified in *Arabidopsis* seedlings [13]. One of them has been identified as 1,2-disinapoylglucose (1,2-DSG). The other DSE was designated as ‘compound 1’ and had been characterized as a disinapoylated monosaccharide, but the precise structure was not elucidated. Because DSEs carry two sinapoyl groups that could potentially mimic sinapyl alcohol during lignin polymerization, the exploration of methods to enhance their production would represent the first step toward their application in the production of a hydrolysable lignin.

**Fig. 1:**
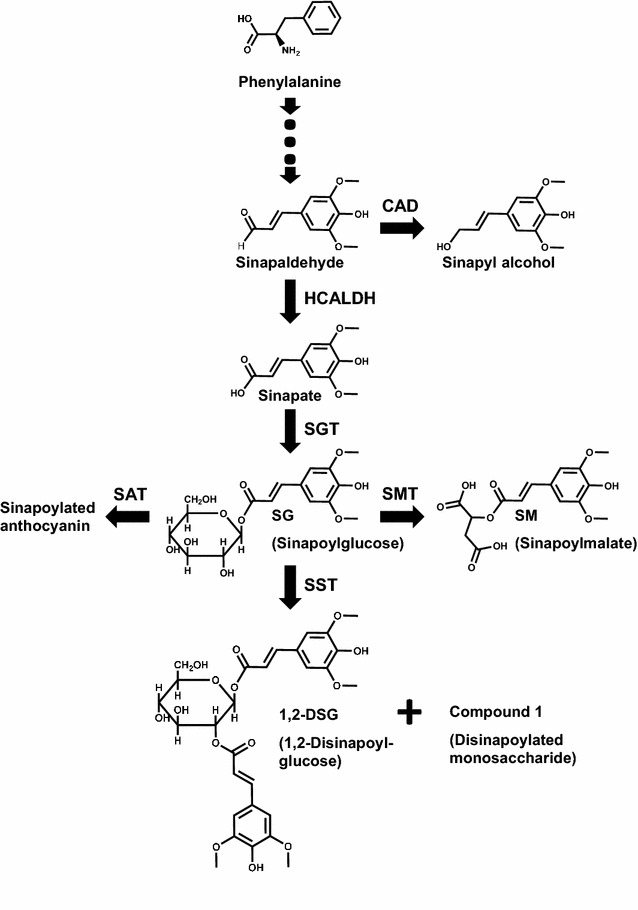
The sinapate ester biosynthetic pathway in *Arabidopsis*. *CAD* cinnamyl alcohol dehydrogenase, *HCALDH* hydroxycinnamaldehyde dehydrogenase, *SGT* sinapic acid: UDPG glucosyltransferase, *SST* sinapoylglucose:sinapoylglucose sinapoyltransferase, *SAT* sinapoylglucose:anthocyanin sinapoyltransferase, *SMT* sinapoylglucose:malate sinapoyltransferase

In *Arabidopsis* leaves, sinapoylglucose (SG) is the common sinapoyl donor in the synthesis of different types of sinapate esters by serine carboxypeptidase-like (SCPL) enzymes (Fig. 1). Sinapoylmalate (SM) and sinapoylated anthocyanins are synthesized by sinapoylglucose:malate sinapoyltransferase (SMT, At2g22990) and sinapoylglucose:anthocyanin sinapoyltransferase (SAT, At2g23000), while 1,2-DSG and compound 1 require sinapoylglucose:sinapoylglucose sinapoyltransferase (SST, At2g23010) for their synthesis [13, 25]. SM and SG are the two major sinapate esters in leaves, whereas the two DSEs are often overlooked because of their low abundance. Here we report that it was possible to increase the amount of DSEs accumulated in *Arabidopsis*, by overexpressing *SST* in a *sinapoylglucose accumulator1*-*6* (*sng1*-*6*) mutant that lacks three SCPL genes, *SMT*, *SAT*, and *SST* [25]. As expected, there was no evidence that these molecules were trafficked to the cell wall, but surprisingly they were instead confined to subvacuolar compartments. This observation reveals an unknown mechanism of phenylpropanoid trafficking, a detailed knowledge of which will be necessary to generate novel forms of hydrolysable lignin in the future.

## Results

### Overexpression of *SST* in *sng1*-*6* increased disinapoyl ester accumulation

**Fig. 2:**
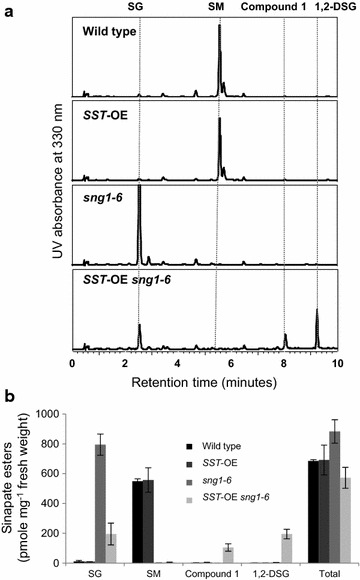
Analysis of soluble metabolites in the 4-week-old leaves. *SG* sinapoylglucose, *SM* sinapoylmalate, *1,2-DSG* 1,2-disinapoylglucose. UV chromatograms at 330 nm (**a**) and sinapate esters content (**b**) in wild-type, *SST*-OE, *sng1*-*6*, and *SST*-OE *sng1*-*6* plants. All leaves harvested from three plants were analyzed independently. *P < 0.05 versus wild type (Student’s t test). Values are mean ± SD

To increase the amounts of DSEs in *Arabidopsis*, we generated transgenic plants overexpressing *SST* under the cinnamate 4-hydroxylase (C4H) promoter [5]. C4H synthesizes *p*-coumaric acid from cinnamic acid, a step that is required to generate all types of monolignols and hydroxycinnamate esters including sinapate esters, and we reasoned that its promoter would be effective in driving transgene expression and in modifying phenylpropanoid metabolic flux in sinapate ester-containing leaves and in lignifying tissues. Among 8 overexpressing lines, we selected two independent lines that displayed higher transgene expression levels compared to the other lines and showed fourfold increase of DSEs at the 7-day-old seedling stages compared to wild type. In these plants, the amount of DSEs was about 10% of total sinapate esters although we did not observe a significant decrease of SM, suggesting that SMT activity still dominates over SST activity even when SST is overexpressed. In order to increase the amounts of DSEs, we crossed the *SST* overexpressing line displaying the highest *SST* expression with *sng1*-*6* [25], a mutant which accumulates SG but which lacks all other known sinapate esters because of a large deletion in a cluster of SCPL genes including *SMT*, *SAT,* and *SST*. The level of SG in *sng1*-*6* was slightly higher compared to the level of SM in wild type and the level decreased by 75% in the presence of *SST* transgene (Fig. 2). Accordingly, the levels of compound 1 and 1,2-DSG increased with their proportions being up to 18 and 34%, respectively, among total sinapate esters.

**Fig. 3:**
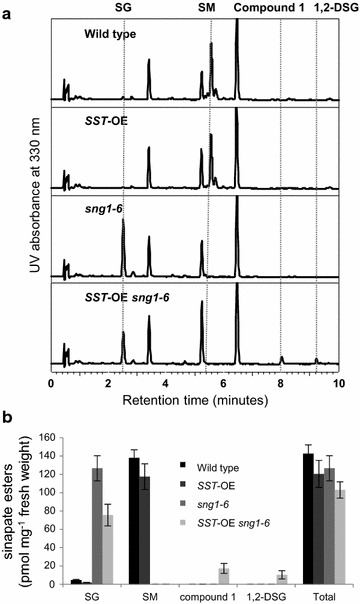
Analysis of soluble metabolites in stems. *SG* sinapoylglucose, *SM* sinapoylmalate, *1,2-DSG* 1,2-disinapoylglucose. UV chromatograms at 330 nm (**a**) and sinapate esters content (**b**) in wild-type, *SST*-OE, *sng1*-*6*, and *SST*-OE *sng1*-*6* plants. Young stems (~10 cm) harvested from three plants were analyzed independently. *P < 0.05 versus wild type (Student’s t test). Values are mean ± SD

Because the stem is the main tissue where lignin is accumulated, we investigated the metabolic engineering effects on the content and distribution of sinapate esters in the stem. When young wild-type stem tissue was analyzed, both SG and SM were present but (Fig. 3a) compound 1 and 1,2-DSG were not detectable. In *sng1*-*6*, SG accumulated, whereas SM was absent as expected [13]. When the *SST* transgene was introduced, SG decreased by 40% while the levels of compound 1 and 1,2-DSG increased to 17 and 10% of the total sinapate ester pool (Fig. 3b), suggesting that SG pool was used to synthesize DSEs.

### Compound 1 was identified as 3,4-disinapoyl-fructopyranose

**Fig. 4:**
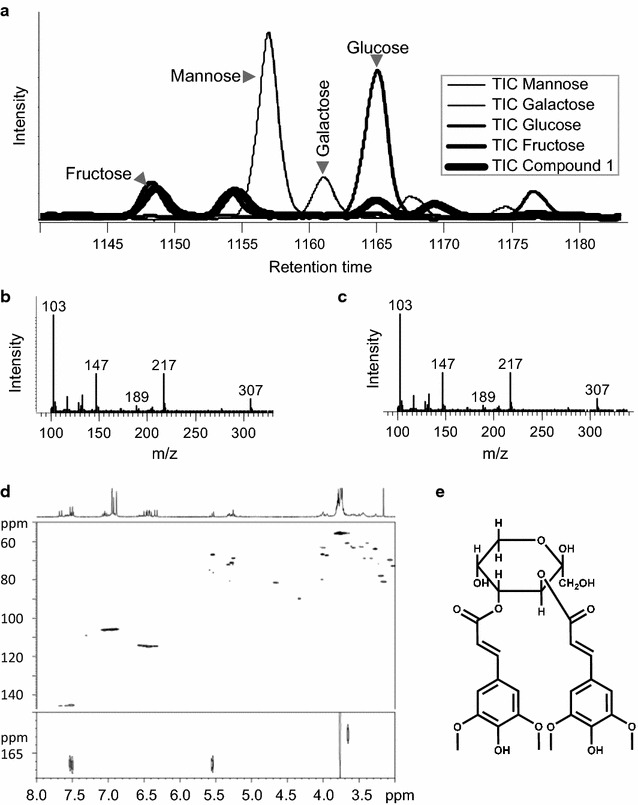
Identification of compound 1. **a** GC chromatograms of mannose, galactose, glucose, fructose, and sugar from compound 1. A major peak of each sugar was indicated. Fructose and sugar from compound 1 co-eluted. **b** GC–MS spectrum of fructose. **c** GC–MS spectrum of the sugar from compound 1. **d** NMR spectrum of compound 1. 1D proton spectrum (*top*); HSQC spectrum (*middle*); HMBC spectrum (*bottom*). **e** Structure of 3,4-disinapoyl-fructopyranose

SST is required to synthesize at least two different sinapate esters, compound 1 and 1,2-DSG. Compound 1 has been reported to be a disinapoylated monosaccharide but the exact structure has not been reported. Using the novel lines generated above, we confirmed the mass of the unknown molecule and further characterized its structure. The UV spectrum of compound 1 displayed the characteristic profile of sinapoylated compounds peaking around 330 nm similar to SG and 1,2-DSG (Additional file 1: Figure S1a). The *m*/*z* value 591 was identified as the largest value on mass spectrums of both 1,2-DSG and compound 1 (Additional file 1: Figure S1b) representing the negative ion form of 1,2-DSG and compound 1 under ESI (−) mode. Following base hydrolysis, the putative sugar residue of compound 1 was derivatized with methoxyamine-HCl and *N*-methyl trimethylsilyl trifluoroacetamide (MSTFA) for GC–MS analyses. As controls, four different kinds of hexoses, glucose, fructose, galactose, and mannose, were processed and analyzed simultaneously. The sugar residue of compound 1 co-eluted with fructose (Fig. 4a), suggesting that the sugar residue is fructose. These chromatograms have two major *m*/*z* peaks at 1148 and 1154, and two minor peaks at 1165 and 1169. On the other hand, mannose, galactose, and glucose displayed one major peak at 1157, 1162, and 1165, and one minor peak at 1168, 1174, and 1177, respectively. Further mass spectrum of compound 1 was identical with that of fructose clearly showing that the sugar residue of compound 1 is fructose (Fig. 4b, c).

For nuclear magnetic resonance (NMR) analyses, after purification by high-pressure liquid chromatography (HPLC) and lyophilization, 0.5–2 mg samples were dissolved in 350 µL d6-DMSO. 1D proton, HSQC, and HMBC spectra were acquired on a Bruker 500 or 800 MHz NMR spectrometer at 25 °C. Due to the potential existence of multiple forms of fructose substructure (alpha- and beta-fructofuranose, and fructopyranose), the most dominant and consistent signals observed in separate sample preparations were assigned to the structure of 3,4-disinapoyl beta-fructopyranose (Fig. 4e).

The proton chemical shifts (in ppm) were observed as 7.50 (2), 6.96 (1), 6.93 (1), 6.46 (2), 5.54 (1), 5.26 (1), 4.01 (1), 3.99 (1), 3.57 (1), and 3.27 (2) (Fig. 4d, upper panel). Carbon chemical shifts (in ppm) were read from HSQC and HMBC as 166.5, 148.0, 146.0, 145.8, 138.5, 124.0, 114.6, 106.0, 97.0, 71.0, 66.8, 66.6, 63.3, and 55.7 (Fig. 4d, medium and lower panel). Lack of high-carbon chemical shifts (>200 ppm) suggests that the fructose exists as a closed form instead of open chain. Lack of clear carbon chemical shift between 80 and 90 ppm, and between 100 and 110 ppm suggests that fructofuranose does not have a dominant population. While carbon chemical shifts at 97 ppm allow us to assign the fructose substructure as fructopyranose, it is not unreasonable to further assume the beta structure as the main form, as our analysis and proposed structure are consistent with the literature report that d-fructose mainly exists in beta-pyranose form in water near room temperature [15]. However, it should be also noted that some sources (e.g., Biological Magnetic Resonance Data Bank) do not appear to have all assignments or have assignments inconsistent with the Funcke paper even for the same fructose model compound.

### Disinapoyl esters are not incorporated into the cell wall at detectable levels

Considering that other SCPL enzymes and sinapate esters are present in the vacuole [21, 46, 49], *SST* presumably synthesizes DSEs in the vacuole where they then accumulate. However, little is known about intra- and extra-cellular trafficking of sinapate esters, leaving open the possibility that some could be incorporated into the cell wall. To test whether this occurs, we prepared cell wall material from *SST*-OE *sng1*-*6* leaves and treated with NaOH to release cell wall-esterified hydroxycinnamic acids. Sinapic acid was not detected in the hydrolysate under these conditions (data not shown). We also measured stem lignin composition and content, using DFRC and the thioglycolic acid method, respectively. However, no significant changes were identified in *SST*-OE *sng1*-*6* stems compared to wild type and *sng1*-*6* stems (Table 1).

**Table 1:** DFRC lignin monomer composition and thioglycolic acid quantification of total lignin

	H (mol%)	G (mol%)	S (mol%)	TGA lignin content (*A*_280nm_ mg^−1^ cell wall)
Col-0	1.20 ± 1.14	83.93 ± 1.89	14.87 ± 1.90	1.58 ± 0.06
*sng1*-*6*	1.96 ± 0.13	81.95 ± 1.20	16.09 ± 1.19	1.55 ± 0.07
*SST*-*OX*	1.25 ± 0.10	80.09 ± 1.45	18.66 ± 1.41	1.52 ± 0.04
*sng1*-*6 SST*-*OX*	1.82 ± 0.20	77.81 ± 2.67	20.37 ± 2.73	1.47 ± 0.05

Values shown are mean ± SD, *A*_280 nm_, absorbance at 280 nm

### A *reduced epidermal fluorescence* phenotype was observed in *SST*-OE *sng1*-*6*

**Fig. 5:**
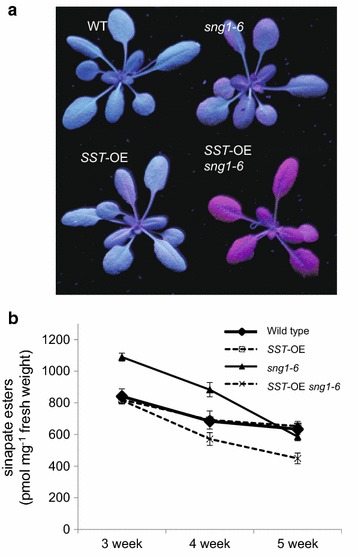
Epidermal fluorescence under UV light (**a**) and total amounts of four major sinapate esters (**b**). Fluorescence was observed in 3-week-old leaves of wild-type, *SST*-OE, *sng1*-*6*, and *SST*-OE *sng1*-*6* plants. All leaves harvested from three plants were analyzed independently. *P < 0.05 versus wild type (Student’s t test). Values are mean ± SD

In *Arabidopsis* leaves examined under UV light, sinapate esters localized in the adaxial epidermal vacuoles emit a blue-green fluorescence. In the absence of sinapate esters, UV can penetrate into the mesophyll and the photosynthetic apparatus it contains, reaching the chlorophylls which fluoresce red. This phenotype has been used to identify the *reduced epidermal fluorescence* (*ref*) series of phenylpropanoid mutants [43]. We used this method to test for changes in soluble sinapate esters in our SST-OE plants. Surprisingly, although blue-green epidermal fluorescence was observed in wild type, *SST*-OE, and *sng1*-*6* plants, we observed a *ref* phenotype in *SST*-OE *sng1*-*6* leaves (Fig. 5a). Generally, this phenotype indicates a reduction of sinapate esters but when we compared the total amount of sinapate esters (SM + SG + 3,4-DSF + 1,2-DSG), no difference was observed between *SST*-OE *sng1*-*6*, wild type, and *SST*-OE in 3-week-old leaves, and only a 16 and 29% reduction were observed in 4- and 5-week-old leaves (Fig. 5b). At both of these stages, the *ref* phenotype was observed in *SST*-OE *sng1*-*6* suggesting that the *ref* phenotype is not simply caused by a reduction in sinapate esters.

**Fig. 6:**
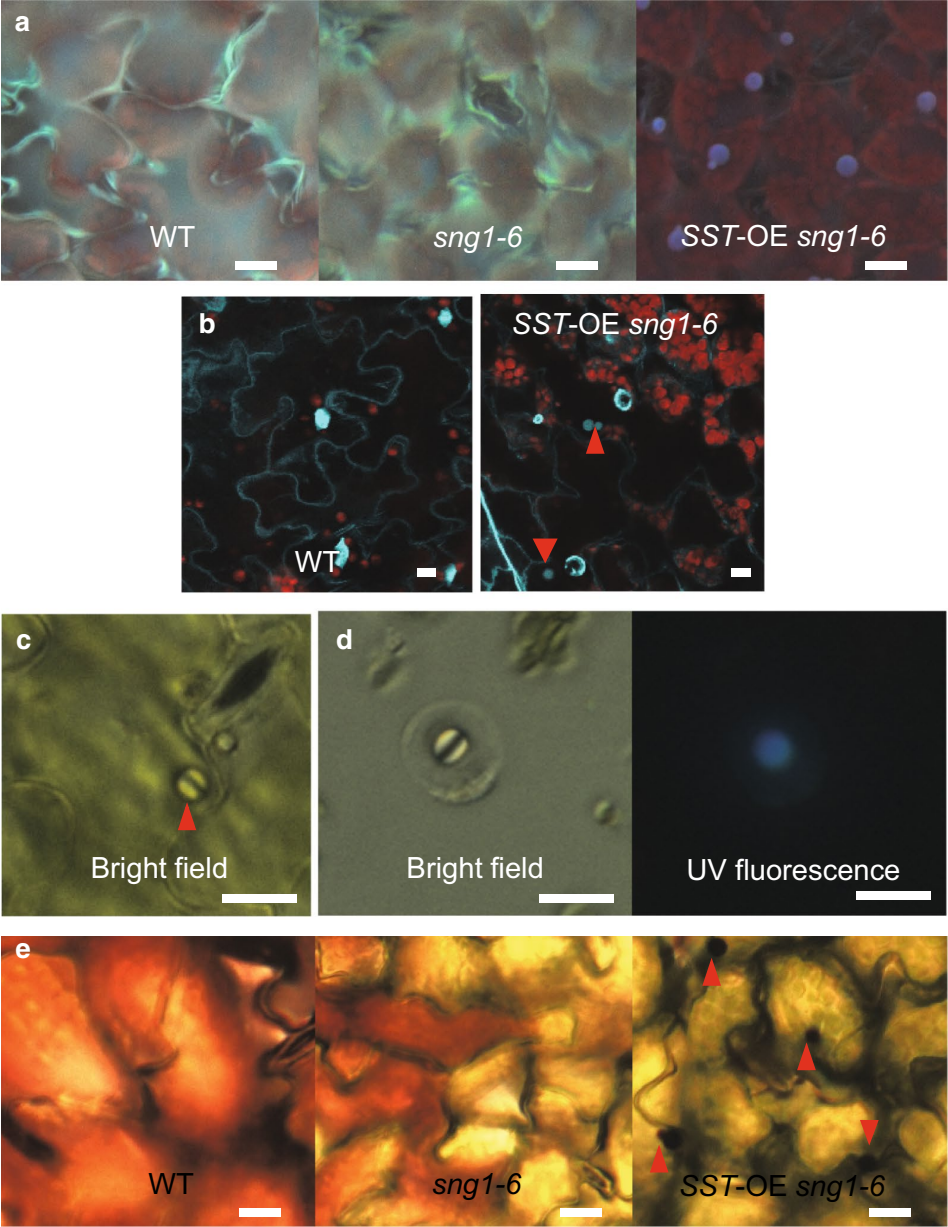
Distribution of fluorescent metabolites in 2- to 3-week-old *SST*-OE *sng1*-*6* plants. **a** UV fluorescence under microscope. **b** Presumptive fluorescent particles (*arrow heads*) after DAPI staining. **c** Fluorescent particles (*arrow heads*) in the adaxial epidermis under bright field. **d** Fluorescent particles in the protoplasts under bright field and UV fluorescence. **e** Particles (*arrow heads*) were stained with neutral *red*. *Scale bars* 10 μm

To understand the *ref* phenotype observed in *SST*-OE *sng1*-*6* leaves, we examined them by UV fluorescence microscopy. In wild type, *SST*-OE, and *sng1*-*6* leaves, fluorescent metabolites were evenly distributed throughout epidermis. In contrast, and unexpectedly, in *SST*-OE, *sng1*-*6* leaves fluorescent inclusions were found (Fig. 6a), suggesting that the cause of the *ref* phenotype in these plants is the result of incomplete shielding of the photosynthetic apparatus by sinapate esters due to their restricted distribution. To check the localization of fluorescent metabolites, leaves were stained with the nucleus-staining dye DAPI. Because of the overlapping emission wavelength between the fluorescent sinapate esters and DAPI, we could not dissect these two types of signals. Nevertheless, only one structure was observed in wild type and its signal was DAPI dependent, but we frequently observed more than one fluorescent structure per cell in *SST*-OE *sng1*-*6* leaves (Fig. 6b), suggesting that fluorescent materials are accumulated in other compartments other than the nucleus. Furthermore, the shape of the DAPI-stained nuclei was not homogenous (Fig. 6b), whereas the fluorescent inclusions were smaller and round (Fig. 6a, b, arrow heads).

We further investigated the localization of the fluorescent inclusions. When the adaxial epidermal layer was peeled, these structures were observed in epidermal cells but not underlying mesophyll cells (Fig. 6c). When protoplasts were isolated, they were observed in the vacuoles of the achlorophylous protoplast from epidermal cells (Fig. 6d). Further, neutral red staining showed that the bodies displayed stronger red signals (arrows in *SST*-*OE sng1*-*6*) compared to the surrounding vacuole (Fig. 6e) suggesting that their lumens are more acidic. This phenomenon was consistently observed in *SST*-*OE sng1*-*6* leaves regardless of staining intensity, which differed depending on leaf position.

### Overall level of sinapate esters increased in the *cad*-*c cad*-*d* mutant

**Fig. 7:**
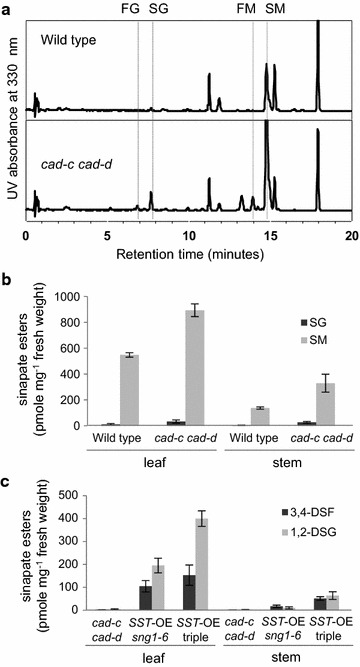
Analysis of soluble metabolites. **a** UV chromatograms of wild-type and *cad*-*c cad*-*d* stems about 10 cm. *FG* feruloylglucose, *SG* sinapoylglucose, *FM* feruloylmalate, *SM* sinapoylmalate. **b** Quantified amounts of SG and SM in 4-week-old leaves and 10 cm stems of wild-type and *cad*-*c cad*-*d* plants. All leaves and young stems harvested from three independent plants were analyzed independently. *P < 0.05 versus wild type (Student’s t test). Values are mean ± SD. **c** Quantified amounts of 3,4-DSF and 1,2-DSG in 4-week-old leaves and stems about 10 cm of *cad*-*c cad*-*d*, *SST*-OE *sng1*-*6*, and *SST*-OE triple (*sng1*-*6 cad*-*c cad*-*d*) plants. All leaves and young stems harvested from three independent plants were analyzed independently. **a**, **b** *P* < 0.05 versus *cad*-*c cad*-*d* and *SST*-*OE sng1*-*6* respectively (Student’s t test). Values are mean ± SD

To further increase the levels of DSEs especially in stems, we explored the idea of introducing the *cad*-*c* and *cad*-*d* mutant alleles [47] into *SST*-OE *sng1*-*6*. Because the final step in monolignol biosynthesis is blocked in *cad*-*c cad*-*d*, precursor aldehydes may be present at higher levels and be redirected to soluble hydroxycinnamate ester pools (Fig. 1). Indeed, our HPLC analyses revealed an increase of SG and SM (respectively 3.0- and 1.6-fold in the leaf and 5.9- and 2.4-fold in the stem) compared to wild type (Fig. 7a, b). Second, feruloylglucose (FG) and feruloylmalate (FM) were detected in *cad*-*c cad*-*d* stems (Fig. 7a), which suggests that a significant amount of coniferaldehyde accumulates in the mutant stems. When we introduced *cad*-*c cad*-*d* into the *SST*-OE *sng1*-*6* background, we observed an even more dramatic accumulation of DSEs. Compared to *SST*-OE *sng1*-*6*, the amounts of 3,4-DSF and 1,2-DSG increased 1.5- and 2.1-fold in leaves and 3.0- and 6.2-fold in stems, respectively (Fig. 7c).

## Discussion

The integration of non-conventional units into lignin in some phenylpropanoid mutants and the successful in vitro incorporation of different phenolics into lignin point to new opportunities for lignin engineering. One promising strategy is to use phenolics with intramolecular ester bonds to generate hydrolysable lignin [7, 36, 52, 54, 56]. In fact, lignins that contain hydrolysable subunits, such as coniferyl ferulate and rosmarinic acid, can be efficiently removed by mild alkaline hydrolysis [16, 51]. Additionally, these novel lignins enhance cell wall saccharification efficiency of hydrolytic enzymes [16, 17]. This increase is observed even without alkaline pretreatment, suggesting that decreased hydrophobicity of lignin or reduced cell wall cross-linking may promote the accessibility of enzymes to the structural polysaccharides of the cell wall. The latter possibility has been reported in artificial lignin polymerized with coniferyl ferulate in the maize cell wall culture system [16]. Moreover, ferulate conjugates were successfully introduced into the poplar cell wall by expressing coniferyl ferulate feruloyl-CoA monolignol transferase, with a concomitant increase in cell wall digestibility [58].

In this study, we evaluated the possibility of engineering enhanced levels of DSEs that could function as hydrolysable lignin subunits. We were able to achieve this goal by overexpressing *SST* in *sng1*-*6*, a genetic background in which competition for sinapoylglucose by SMT was eliminated. The introduction of *cad*-*c cad*-*d* double mutant into *SST*-OE *sng1*-*6* further increased DSE concentration. This effect was more dramatic in stems compared to leaves possibly because of the involvement of CAD in lignification which occurs to a greater extent in stems and might, therefore, provide more DCS precursors if blocked.

To generate hydrolysable lignin using DSEs or any other atypical lignin precursor, it is essential that they be appropriately located to and within lignifying tissue. Our localization analyses showed that DSEs are instead localized in subvacuolar particles, at least in leaves. Similar particles have been identified in the vacuoles of more than 70 species as anthocyanin-containing particles called anthocyanoplasts [33] or anthocyanic vacuolar inclusions (AVIs) [11, 31]. AVIs from the *Lisianthus* epidermis have been characterized as protein-anthocyanin complexes and are preferentially associated with four cyanidin and delphinidin acylated 3,5-diglycosides [31]. AVIs from grapevine (*Vitis vinifera* L.) show preferential binding with acylated anthocyanins compared to non-acylated forms [11]. Recently, AVIs were also observed inside the large central vacuole of *Arabidopsis* cells, possibly as membrane bound bodies [34, 35]. The formation of AVIs correlates with the accumulation of specific types of anthocyanins and cyanidin 3-glucoside (C3G) and its derivatives, rather than the total amount of anthocyanins, and AVI formation is defective in autophagy mutants [34]. These observations have led to the hypotheses that C3G may induce autophagy to generate subvacuolar particles or that C3G may stabilize autophagosomes that are normally formed in the vacuolar lumen where they are degraded by hydrolytic enzymes. Based on our neutral red staining results, DSE-containing bodies are more likely to be membrane bound rather than protein-DSE complexes. Like AVIs in *Arabidopsis*, neutral red strongly stained these particles compared to the vacuolar lumen suggesting that a more acidic environment is formed in these AVIs. The same concepts may explain the formation of the DSE particles. If specific anthocyanins are found within AVIs in *Arabidopsis*, the same may be true for sinapate esters: the formation of these bodies may depend on the type rather than the amount of sinapate esters. For example, the fact that DSEs have two sinapoyl groups may allow these compounds to coat autophagosomes to stabilize their structure or protect them degradation. If true, the formation of these bodies would be a consequence of the specific chemical properties of DSEs.

Monolignol biosynthetic enzymes are located in the cytosol [1, 9, 42, 44], and monolignols must therefore either diffuse through the plasma membrane or be captured by transporters for secretion into the cell wall. Recently, an ABC-like transporter *AtABCG29* has been identified as a plasma membrane cinnamyl alcohol transporter in *Arabidopsis* [2]. There are 12 close homologs of AtABCG29 that may transport not only the other two conventional units but also related analogs. For example, other monolignol intermediates such as ferulate, coniferaldehyde, and sinapaldehyde are also synthesized in cytosol and may be accessible to these transporters. Coniferyl ferulate may be similarly transported [58]. This may be the reason why these units can be integrated into lignin [4, 20, 37, 47]. On the other hand, sinapate esters including DSEs are thought to be synthesized in the vacuole where SCPL enzymes such as SMT are localized [21, 46, 49]. If sequestered within the vacuole and particularly within vacuolar inclusions, DSEs will not be accessible to ABC-like transporters nor will they be integrated into lignin.

Even though DSEs are localized inside the vacuole, they might be transported to the cell wall via mechanisms that have been suggested to explain the mobilization of monolignol glucosides for lignification in certain species [28]. Coniferin was first detected in the xylem of gymnosperms where it serves as a supply of monolignols following activation of cambial activity in the spring [26, 45, 50]. These data suggest that it is possible to mobilize vacuolar reserves to support lignification, but given that monolignol glucosides are thought to be present primarily in gymnosperms, and these mechanisms may not be present in species of interest for biofuel production. As a result, the mechanisms that sequester potential hydrolysable lignin subunits to the vacuole, and the machinery that might mobilize these vacuolar pools to the apoplast are clearly a topic of great importance as we consider attempts to engineer hydrolysable lignins in biofuel crop species.

## Conclusions

Although we successfully upregulated the accumulation of the target DSEs, we did not find any evidence showing the integration of DSEs into the cell wall, and furthermore, they were instead sequestered in bodies within the vacuole. Our results suggest that although phenylpropanoid metabolic engineering for the purposes of lignin modification is possible, a deeper understanding of sequestration and transport mechanisms will be necessary for successful lignin engineering through this route, at least in the context of a subset of these possible lignin modification molecules.

## Methods

### Plant material and growth conditions

*Arabidopsis thaliana* Columbia-0 was used as the wild type for all experiments. T-DNA insertion lines were obtained from ABRC stock center (*cad*-*c*: SAIL_1265_A06; *cad*-*d*: SAIL_776_B06). Plants were grown in Redi-earth Plug & Seedling Mix (Sun Gro Horticulture, Vancouver, British Columbia, Canada) and supplied with Scotts Osmocote Plus controlled release fertilizer (Hummert International, Earth City, MO, USA) at 22 °C under a 16-h light/8-h dark photoperiod.

### Vector construction for overexpression

*SST* gDNA fragments were amplified with CC2194 (5′-ggg gac aag ttt gta caa aaa agc agg ctt cgc tga aag gac tca aat c-3′) and CC2195 (5′-ggg gac cac ttt gta caa gaa agc tgg gtg tta tga caa gga gac aaa gga ca-3′) using Columbia wild-type genomic DNA as a template. The Gateway cloning system was employed to construct a binary vector for plant transformation using pCC1155 as a pDONR vector and pCC0996 as a destination vector as described [57].

### HPLC analyses to quantify soluble metabolites

Plant leaf and stem tissues were ground in 1.5 mL microcentrifuge tubes in liquid nitrogen, extracted with 50% methanol (*v*/*v*) (10 µL mg^−1^ fresh weight) for 30 min at 65 °C, and centrifuged (11,000*g*, 25 °C, 10 min). Soluble metabolites were separated using a Shimadzu (www.shimadzu.com) Shim-pack XR-ODS column (2.2 µm particle size, 75 × 3.0 mm internal diameter) with a flow rate of 1 mL min^−1^. The mobile phase consisted of eluent A (0.1% formic acid in water) and eluent B (0.1% formic acid in acetonitrile). Samples (10 µL) were injected into the starting eluent composed of 90% A/10% B. After 0.5 min, the proportion of B was increased linearly to 20% over 6.5 min, then to 35% over 5 min, then to 95% over 0.5 min, and held at 95% for 1 min. To ensure separation of soluble metabolites in the stem tissue as shown in Fig. 7, a longer method with a flow rate of 0.8 mL min^−1^ was employed. Samples (10 µL) were injected into the starting eluent composed of 90% A/5% B. After 0.3 min, the proportion of B was increased linearly to 25% over 29.7 min, then to 95% over 1 min, and held at 95% for 1 min.

### Microscopic observation of sinapate ester distribution

To observe the distribution of sinapate esters in the leaf epidermis, a fluorescence microscope (Leica DMR) equipped with a band path filter BP 340-380, dichroic mirror 400, and long path filter LP 425 was used. For neutral red (NR) staining, whole leaves were incubated for 20 min in NR solution (1 mg mL^−1^ in water) and washed briefly in water before observation using a microscope (Leica DMR). For DAPI staining analysis, confocal imaging was performed using Nikon A1R MP with Apo LWD 40X 1.15 water emersion objective (filter 1: excitation 408 nm, emission 425–475 nm, filter 2: excitation 640 nm, emission 575–625 nm, pinhole: 26.8 µm) after incubating for 30 min in 0.1 M sodium phosphate buffer (pH 7), 1 mM EDTA, 0.1% Triton X-100 (*v*/*v*), and 0.5 µg mL^−1^ DAPI.

### LC–UV–MS and GC–MS analyses to identify compound 1

Sinapoylated compounds were purified using semi-preparative HPLC and were further analyzed on an Agilent 1100 system (Palo Alto, CA). Samples (10 µL) equipped with a Shimadzu Shim-pack XR-ODS column (2.2 µm particle size, 75 × 3.0 mm internal diameter) and diode array detector to collect UV spectra between 210 and 400 nm. The eluent was then introduced into an Agilent MSD-TOF spectrometer in negative mode electrospray ionization mode (capillary voltage: 3.2 kV, nebulizer gas pressure: 55 psig, gas temperature: 350 °C, drying gas flow rate: 11 L min^−1^, fragmentor voltage: 125 V, skimmer: 60 V, and OCT RF: 250 V). A mass range of 80–1000 *m*/*z* was scanned and Agilent’s reference mass correction solution was used as a lock mass to ensure mass accuracy. Data was acquired and analyzed using Agilent’s MassHunter software (v. B.03).

GC–MS analysis was performed using a Pegasus 4D gas chromatography/gas chromatography time-of-flight mass spectrometer (GCxGC/TOF–MS, LECO Corporation, St. Joseph, MI). With a 20:1 split ratio, samples (2 µL) were injected and run through the DB-5MS capillary column (J&W Scientific, 30 m × 0.25 mm × 0.25 µm) along with high purity carrier gas helium (1.2 mL min^−1^). The temperature program began at 50 °C with a hold time of 0.2 min^−1^, increased at a rate of 10 °C min^−1^ to 250 °C with a hold time of 10 min, and then increased at a rate of 25 °C min^−1^ to 300 °C with a hold time of 5 min. The injection inlet and mass spectrometer transfer line temperatures were held at 280 °C. The electron impact (EI) ion source was held at 200 °C, with a filament bias of 70 eV. Mass spectra were collected from 100 to 600 *m*/*z* at 40 spectra/s.

### NMR analyses to identify compound 1

Compound 1 collected using semi-preparative HPLC was further purified using Shimadzu Shim-pack XR-ODS column (2.2 µm particle size, 75 × 3.0 mm internal diameter). After drying, about 0.5 to 2 mg of the compound was dissolved in DMSO and NMR data (1D H, HSQC and HMBC) was acquired at 25 °C on a Bruker Avance 800 or 500 MHz spectrometer equipped with a triple resonance gradient probe.

### Analysis of cell wall-bound hydroxycinnamic acids

Cell wall material was prepared from 4-week-old leaves using a modified method based on [32]. Plant material was ground with liquid nitrogen and washed with 0.1 M sodium phosphate buffer at 65 °C, then with 70% ethanol (*v*/*v*) at 80 °C five times, and then with acetone. The samples were then air dried and 20 mg of the cell wall was mixed with 500 µL of 1.0 M NaOH and 20 µL of 0.1 M 3,4,5-trimethoxy cinnamic acid as an internal standard and incubated with shaking for 1 day. Samples were then acidified with 100 µL concentrated HCl. After centrifugation (11,000*g*, 25 °C, 20 min), hydrolysis products were extracted by vortexing 500 µL of the supernatant with 1 mL ethyl acetate. After separation of the two phases, the upper phase was dried in a speed vacuum concentrator, and redissolved in 50 µL of 50% methanol (*v*/*v*) for HPLC analysis. The same Shim-pack XR-ODS column and mobile phases used for soluble metabolites was used with a flow rate of 1 mL min^−1^. Samples (20 µL) were injected into the starting eluent composed of 95% A/5% B. After 0.3 min, the proportion of B was increased linearly to 20% over 0.1 min, then to 12.6% over 6.1 min, then to 70% over 5.5 min, then to 95% over 1 min, and held at 95% for 1 min.

### Lignin content and composition analysis

Eight-week-old inflorescence stems were cut into small pieces and finely ground using mixer mill (Retsch Mixer Mill MM400). Ground tissue was washed with 0.1 M sodium phosphate buffer (pH 7.2) at 50 °C for 30 min, extracted seven times with 70% ethanol (*v*/*v*) at 80 °C for 15 min, and washed twice with 100% acetone for 10 min. The cell wall residue was dried at room temperature overnight. Thioglycolic acid quantification of lignin and DFRC lignin analysis were performed as previously reported [6, 29].

## Additional file

**Additional file 1: Figure S1.** UV spectra (a) and MS spectra under ESI (-) mode (b) of 1,2-DSG and compound 1.
