# Supplementary figures and images for: Genetic engineering of Arabidopsis to overproduce disinapoyl esters, potential lignin modification molecules
Source: Biotechnol Biofuels. 2017 Feb 17;10:40. doi: 10.1186/s13068-017-0725-0 (PMC5316160; doi:10.1186/s13068-017-0725-0)

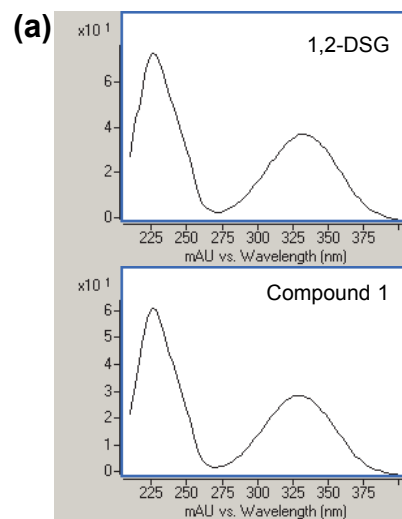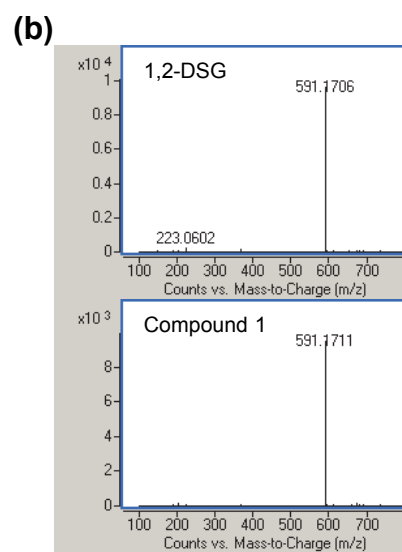

Supplement: Supplementary file 1 — Additional file 1: Figure S1. UV spectra (a) and MS spectra under ESI (-) mode (b) of 1,2-DSG and compound 1. [file 13068_2017_725_MOESM1_ESM.pdf]
